# Bilateral Iliosacral and Transsacral Screws Are Biomechanically Favorable and Reduce the Risk for Fracture Progression in Fragility Fractures of the Pelvis—A Finite Element Analysis

**DOI:** 10.3390/bioengineering12010027

**Published:** 2025-01-01

**Authors:** Moritz F. Lodde, Matthias Klimek, Elmar Herbst, Christian Peez, Oliver Riesenbeck, Michael J. Raschke, Steffen Roßlenbroich

**Affiliations:** Department of Trauma, Hand and Reconstructive Surgery, University Hospital Münster, 48149 Münster, Germany

**Keywords:** FE analysis, biomechanics, SI screw, transsacral SI screw, fragility fracture of the pelvis (FFP), fracture progression of FFP (FP)

## Abstract

(1) Background: The incidence of fragility fractures of the pelvis (FFP) has increased significantly over the past decades. Unilateral non-displaced fractures, defined as FFP II, are the most common type of fracture. When conservative treatment fails, surgical treatment is indicated. We hypothesize that the use of bilateral SI screws (BSIs) or a transsacral screw (TSI) is superior compared to a unilateral screw (USI) because of a significant reduction in the risk of adjacent fractures and a reduction in fracture progression. (2) Methods: A finite element model of a female pelvic ring was constructed. The ligaments were simulated as tension springs. The load was applied through the sacrum with the pelvis fixed to both acetabula. An FFP IIc was simulated and fixed with either a USI or BSI or TSI. The models were analyzed for a quantitative statement of stress and fracture dislocation. (3) Results: The BSI and TSI resulted in less dislocation compared to the USI. The stress distribution on both sides of the sacrum was favorable in the BSI and TSI groups. The BSI resulted in a higher rotational stability compared to the TSI. (4) Conclusions: The use of either a BSI or TSI for fixation of unilateral FFP is biomechanically favorable compared to the use of a USI. In addition, the use of a BSI or TSI reduces the stress on the contralateral uninjured side of the sacrum. This may reduce the risk of an adjacent fracture or fracture progression.

## 1. Introduction

Clinically, fragility fractures of the pelvis (FFP) are associated with a high one-year mortality rate, ranging from 11–27% and a substantial loss of quality of life or function [1,2,3,4]. FFP result from low-energy falls or occur spontaneously. These fragility fractures are associated with a reduced bone quality or osteoporosis [5]. FFP II—unilateral non-displaced fractures of the anterior and posterior pelvic ring—are the most commonly observed fracture type [6,7,8,9,10]. The results of conservative and the surgical treatment techniques for FFP II are controversially discussed and reported in the literature [8,9,11]. When conservative treatment fails, surgical treatment is indicated [8,9,11,12]. While several other techniques have been described in the literature, the SI screw is widely accepted as the standard of care treatment [13,14,15,16,17]. However, several biomechanical studies have been conducted to further improve the fixation of the posterior pelvic ring using percutaneous SI screws [18,19,20]. A recently published systematic review and meta-analysis showed that fracture progression is relatively high in patients with FFP [17,21]. The authors suggested that a TSI could reduce the risk of fracture progression [17].

Finite element analysis (FE analysis) is a well-established method for investigating the biomechanics of different percutaneous screw fixation techniques for unstable pelvic ring fractures, even allowing for the simulation of bone cement [22,23,24,25,26,27,28,29,30,31,32].

The aim of the present FE analysis was to investigate the biomechanical behavior of a USI, BSI, and TSI for the fixation of FFP IIc in terms of fracture dislocation and stress distribution.

We hypothesize that the use of bilateral SI screws (BSIs) or a transsacral screw (TSI) in unilateral fractures of the pelvic ring is biomechanically favorable compared to a unilateral SI screw (USI). We further hypothesize that the risk of fracture progression is reduced with a BSI and a TSI.

## 2. Materials and Methods

A finite element model of a female pelvic ring was created using the CT data from the Visible Human Project. The model was segmented in Slicer 3D [33].

Segmentation was performed with a threshold between 220 and 2000 Hounsfield units (HU) based on the CT scan [34].

Voxels outside the pelvic ring were deleted, and the segmentation was smoothed. The surface of the segmentation was equalized to a faceted model with 0.7 mm triangles to reduce the extrusions from the segmentation while still smoothing the surface and maintaining the computational complexity. This model was transformed into a full-body model. On the full-body model, the surface for each ligament insertion point was manually created. The symphysis and articular disc were manually created as an automatically attached body to the bone surface. The pelvic ring was meshed using linear tetrahedron elements.

For the convergence analysis, the pelvic ring was fixed at the upper level of the sacrum, and a bipedal stance was simulated.

For the mesh analysis, the change in the maximum tension and compression was used as the reference. A change of less than 5% was considered accurate. At this stage, the bone was modeled as a linear elastic material with a Young’s modulus of 6000 megapascals (MPa). After convergence analysis, a maximum size of 5 mm and a minimum size of 1 mm were chosen for the tetrahedrons to ensure sufficient accuracy and speed of execution.

The resulting mesh was imported into bonemat (BIC Lab, Bologna, Italy). In bonemat, each element was assigned an individual Young’s modulus based on the CT scan [35]. The maximum HU value was sorted by the apparent density $\rho_{app}=1.8\;g/{\mathrm{cm}}^{3}$ [36]. The Dalstra formula was used to correlate the apparent density. Values of Young’s modulus, E, were determined as follows [37]:

(1) $$\rho_{app}=0.00032362*HU+1$$

(2) $$E=1958.6*{\rho_{app}}^{2.33}$$

The Young’s modulus values ranged from 200 MPa to 6530 MPa, simulating the bone conditions of an elderly woman [32]. The range was divided into increments of 50 MPa. A total of 1266 different materials were defined and processed in the mesh. After importing into Ansys Mechanical (v24.1, Ansys, Inc., Canonsburg, PA, USA), contacts between the cartilage and bone were determined as boned.

The load was applied to the first sacral body of the sacrum. The pelvis was fixed to both acetabular bones. The load value corresponded to the validation of Miller et al. [38].

A load of 294 N was applied in the anterior, posterior, superior, and inferior directions. Sacral motion and the validation parameters were measured. In the present study, the ligament configurations from the previously published study by Eichenseer et al. were used for all ligaments except the superior pubic ligament (SP) and inferior pubic ligament (IP) (Table 1) [31]. The values used for SP and IP were taken from the study by Shi et al. [24].

**Figure 1:**
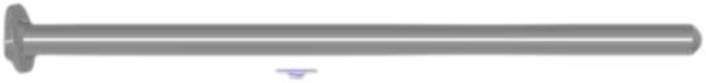
This figure shows the SI screw with a washer used to simulate the USI, BSI, and TSI.

The next step was to simulate an FFP IIc [12]. The SI screw insertions were placed by a board-certified trauma surgeon and independently validated by another board-certified trauma surgeon. The design of the USI, BSI, and TSI was adapted from commonly used implants (Marquardt Aaxomed, ISG System, Freiburg, Germany). The screws were simulated without threads and bonded to the bone (Figure 1).

A maximum walking force of 2048 N was applied separately in each acetabulum during the single-legged stance, and consequently, half of the maximum force was applied separately during the bipedal stance [39].

Normal stress and fracture dislocation were investigated in the bone aligned with the anatomical directions, taking into account that the von Mises stress is only applicable to ductile material and bone behavior is brittle. All the different fixation techniques were analyzed in order to make a quantitative statement about the stresses and dislocations in the sacrum. In this way, it was possible to assess the load on the uninjured contralateral part of the sacrum. Stress and dislocation were measured at the posterior pelvic ring at the superior and inferior fracture gap in zone II according to the classification of Denis et al. [40]. Dislocation at the anterior pelvic ring was measured at the fracture gap at the superior pubic ramus.

## 3. Results

**Figure 2:**
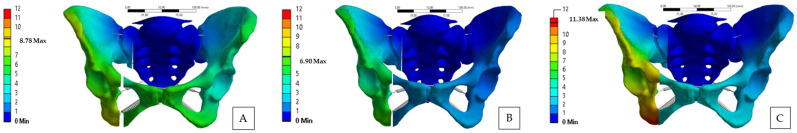
This figure shows the dislocation [mm] in the simulated FFP IIc. The load was applied via a bipedal stance. (**A**) Fixation with the USI. (**B**) Fixation with the BSI. (**C**) Fixation with the TSI. Dislocation of the fracture at the posterior pelvic ring was less with the BSI or TSI compared to the USI. Dislocation at the anterior pelvic ring was less with the BSI compared to the TSI or USI.

**Figure 3:**
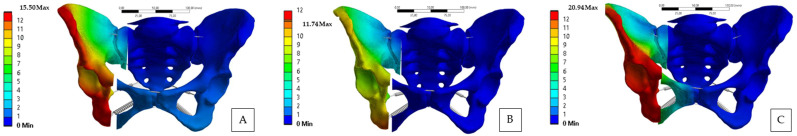
This figure shows the dislocation [mm] in the simulated FFP IIc. The load was applied via a right one-legged stance. (**A**) Fixation with the USI. (**B**) Fixation with the BSI. (**C**) Fixation with the TSI. Dislocation at the posterior pelvic ring was less with the TSI compared to the BSI and USI. Dislocation at the anterior pelvic ring was less with the BSI.

**Figure 4:**
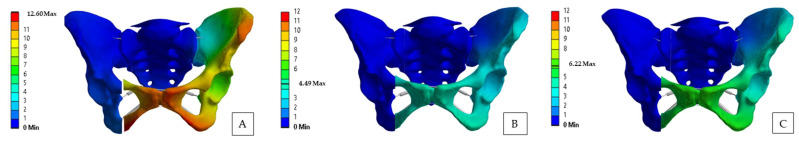
This figure shows the dislocation [mm] in the simulated FFP IIc. The load was applied via a left one-legged stance. (**A**) Fixation with the USI. (**B**) Fixation with the BSI. (**C**) Fixation with the TSI. Dislocation was less with the BSI. Dislocation was less with the TSI compared to the USI. Dislocation at the anterior pelvic ring was less with the BSI.

**Figure 5:**
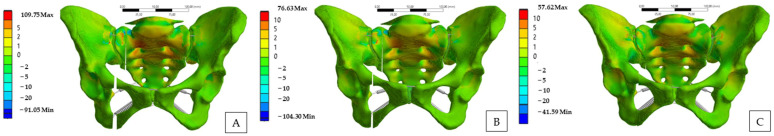
This figure shows the normal stress [MPa] in the mediolateral direction in the simulated FFP IIc. The load was applied via a bipedal stance. (**A**) Fixation with the USI. (**B**) Fixation with the BSI. (**C**) Fixation with the TSI. The observed stress at the posterior pelvic ring was less with the BSI or TSI. The stress at the anterior pelvic ring was less with the BSI compared to the TSI or USI.

**Figure 6:**
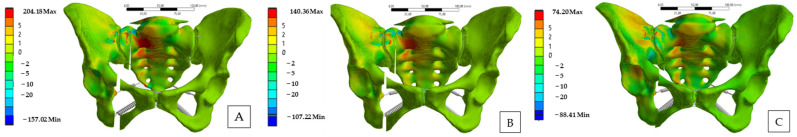
This figure shows the normal stress [MPa] in the mediolateral direction in the simulated FFP IIc. The load was applied via a right one-legged stance. (**A**) Fixation with the USI. (**B**) Fixation with the BSI. (**C**) Fixation with the TSI. The observed stress at the posterior pelvic ring was less with the TSI compared to the BSI or USI. The stress at the anterior pelvic ring was less with the BSI.

**Figure 7:**
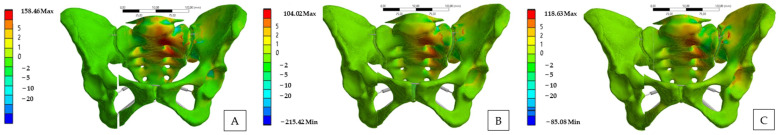
This figure shows the normal stress [MPa] in the mediolateral direction in the simulated FFP IIc. The load was applied via a left one-legged stance. (**A**) Fixation with the USI. (**B**) Fixation with the BSI. (**C**) Fixation with the TSI. The stress was less with the BSI or TSI compared to the USI. The stress on the anterior pelvic ring was less with the BSI.

**Figure 8:**
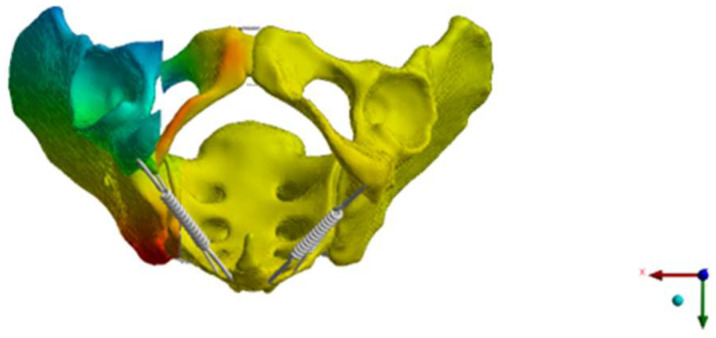
This figure shows, from a more inferior view, that the fracture gap in the inferior–superior direction was larger with the TSI.

The BSI and TSI provided more stability and less dislocation compared to the USI (Figure 1, Figure 2, Figure 3, Figure 4, Figure 5 and Figure 6, Table 2). The fracture gap at the posterior pelvic ring was lowest with the TSI (0.69 mm) compared to the BSI (1.21 mm) and USI (1.84 mm). The stress distribution on both sides of the sacrum was favorable with the BSI and TSI. It should be noted that the TSI was even more stable than the BSI. The posterior dislocation was less with the TSI (Table 2). However, the BSI resulted in a smaller fracture gap distance in the inferior–superior direction compared to the TSI (Figure 2, Figure 3, Figure 4, Figure 5, Figure 6 and Figure 7). Figure 8 shows the increased fracture gap distance in the inferior–superior direction with the TSI from a more inferior view for better visualization. When the load was applied via a bipedal stance, the distance of the fracture gap at the anterior pelvic ring was 1.53 mm with the BSI and −0.20 mm with the TSI (Table 2). With the TSI, the medial and lateral parts of the superior pubic ramus slid over each other (Figure 2, Figure 3, Figure 4, Figure 5, Figure 6, Figure 7 and Figure 8). The same was true when the load was applied via the right-sided one-legged stance (fracture gap distance: 3.31 mm for BSI vs. −1.19 mm for TSI, Table 2).

The present FE analysis shows that the use of a USI to fix a unilateral FFP IIc led to a higher stress concentration on the contralateral uninjured side of the sacrum (Figure 2, Figure 3, Figure 4, Figure 5, Figure 6 and Figure 7, Table 3). This was true for both the single-legged and bipedal stance (Table 3). The BSI and TSI had similar stress distributions (Table 3). The highest stress concentration was observed at the uninjured side of the sacrum in the simulated single-legged stance of the uninjured side (stress: USI at S1: 15.71 MPa, BSI at S1: 9.98 MPa, TSI at S1: 8.91 MPa) (Figure 2, Figure 3, Figure 4, Figure 5, Figure 6 and Figure 7, Table 3). Correspondingly, the BSI and TSI resulted in a lower stress concentration on the contralateral side of the sacrum.

## 4. Discussion

The present study focused on different types of fixation of FFP IIc and the risk of an adjacent fracture of the uninjured contralateral part of the sacrum. The main findings of the present study are that the BSI and TSI were biomechanically favorable compared to the USI in terms of unilateral fragility fractures of the pelvis (FFP IIc). Furthermore, the BSI and TSI reduced the risk of an adjacent fracture of the contralateral uninjured side of the sacrum. The TSI was even more stable than the BSI. However, the BSI resulted in higher rotational stability compared to the TSI. Our hypotheses were confirmed.

In the present study, an FFP IIc was simulated [12]. According to the comprehensive classification of fragility fractures of the pelvic ring, FFP II are defined as non-displaced fractures [12]. FFP IIa are isolated non-displaced fractures of the posterior pelvic ring without involvement of the anterior pelvic ring. FFP IIb are defined as non-displaced sacral fractures combined with anterior disruption [12]. More specifically, FFP IIb are pubic and ischial rami fractures combined with a crush zone of the sacral ala without displacement [12]. In contrast to this, FFP IIc lesions are pubic and ischial rami fractures combined with a non-displaced sacral ala fracture [12]. These subtle differences are important for the results of the present study because FFP IIc are more unstable than FFP IIa and FFP IIb. In particular, the rotational instability is higher in FFP IIc compared to FFP IIa and FFP IIb.

As percutaneous SI screw fixation has become widely accepted as the standard of care, the type of fracture and, thus, the degree of instability and the rotational instability influence the risk of screw loosening [19]. The present study shows that TSIs and BSIs are superior compared to USIs. TSIs result in a smaller fracture gap distance and less stress on the posterior pelvic ring compared to BSIs. TSIs may prevent screw loosening and reduce pain better than BSIs. Biomechanical studies investigating this issue are lacking. The distance of the fracture gap in the inferior–superior direction at the anterior pelvic ring is smaller with BSIs. The use of BSIs with slightly different axes provides greater rotational stability. This is consistent with another biomechanical study analyzing a screw-in-screw fixation for fragility fractures of the pelvis [19].

Adjacent fractures have long been recognized in the field of osteoporotic vertebral compression fractures. Data on adjacent fractures of the sacrum are lacking in the literature. However, a recently published systematic review and meta-analysis showed that fracture progression is relatively high in patients with an FFP [17,21]. Sensitivity analysis showed that the pooled prevalence of fracture progression in patients with an FFP was 10% (95% CI, 4–18%) [17]. A pooled prevalence >10% is relevant [41]. Yamamoto et al. suggest that fixation of a unilateral sacral fracture with a TSI may prevent subsequent fracture progression of the contralateral side of the sacrum [17]. The results of the present study support this previously published hypothesis. Clinically, the use of BSIs and TSIs is safe and feasible [16,42,43]. In FFP IIc, BSIs and TSIs are biomechanically superior due to a higher overall construct stability, a better load distribution, and a reduced risk of an adjacent fracture and fracture progression, respectively [17]. The stress and strain on the contralateral side of the sacrum are particularly high when the uninjured side is loaded. This emphasizes the need for BSIs or TSIs, as elderly patients may not be able to comply with post-operative weight-bearing restrictions [44,45]. In their retrospective study, Heydemann et al. showed that TSIs or USIs had no positive or negative effect on pain and functional outcome at a minimum follow-up of one year [46]. However, young patients were also included, and the mean ISS (Injury Severity Score) was 23.4 and 23.6, respectively. In addition, TSIs are generally likely to be used in patients with unstable injuries or in osteopenic patients [46]. Therefore, these inclusion criteria and methods may explain the findings. A clinical trial comparing USIs and TSIs or BSIs for the fixation of FFP is lacking.

Several biomechanical studies and FE analyses have examined fixation techniques of the posterior pelvic ring after high-energy trauma. The more distant and parallel the implant used is to the sacroiliac axis of rotation, the more favorable the fixation [25]. Zhang et al. found in simulated Tile B and C pelvic ring injuries that a single SI screw in S1 (first sacral body) was sufficient to stabilize type B injuries and that an additional SI screw in S2 (second sacral body) increased the biomechanical stability [26]. In another FE analysis, Wu et al. investigated six different combinations of SI screws and TSIs for the fixation of Tile B or C pelvic ring fracture models (Dennis-II-type fracture) [27]. In their study, the highest stability was achieved with an oblique SI screw in S1 and a TSI in S2 [27]. In a simulation of a Tile C pelvic ring fracture, Hu et al. showed that stress shielding was favorable with the fixation of two SI screws and a minimally invasive adjustable plate [29]. In a simulated bilateral Tile C pelvic ring fracture, the risk of fracture was low with bilateral symmetric double-segmental screws [28]. The authors strongly recommend the use of bilateral symmetric screw fixation [28]. Ma et al. observed in a unilateral Tile C pelvic ring fracture that the risk of screw breakage was lower with double-segmental screw fixation than with single-segmental screw fixation [30]. The studies published to date have evaluated fixation techniques for Tile B and Tile C pelvic ring fractures following high-energy trauma [26,27,28,29,30]. The main findings are that bilateral fixation techniques or the use of an additional screw result in increased biomechanical stability and a favorable stress distribution. These findings are consistent with the results of the present study.

A biomechanical study investigating the competence of a TSI and USI for the fixation of an FFP IIc showed that the TSI resulted in a significantly higher stability for the gap angle, flexion, vertical motion, and overall stability compared to the USI [18]. The authors suggest that TSIs should be used clinically for the fixation of FFP IIc [18]. These findings and conclusions are again consistent with the present study.

Strengths and limitations: The present FE analysis contains known approximations and limitations that are comparable to the limitations of previously published studies [26,27,28,29,30]. The FE analysis presented is an in vitro model. In the present model, the effects of the ligaments were taken into account. The effects of the pelvic muscles, fascia, and organs on pelvic stability were not considered.

All FE analyses in the present study were performed under the same in vitro experimental conditions. Biomechanical differences between the different fixation techniques could be reliably investigated without the confounding factors that occur in cadaveric biomechanical testing. Another advantage of the present study is the global anistropic bone behavior and the use of a hyperelastic symphysis. The pelvic ring was meshed using linear tetrahedral elements. Due to the complex design of the bone, tetrahedral elements are less rigid and more accurate in modeling the bone surface than hexahedral elements.

In conclusion, BSIs and TSIs are biomechanically advantageous for unilateral FFP IIc fixation. BSIs and TSIs may reduce the risk of FFP fracture progression, leading to a better clinical outcome. Further clinical studies analyzing the prevention of fracture progression are needed.

## 5. Conclusions

The use of BSIs or TSIs for the fixation of unilateral FFPc is biomechanically favorable compared to the use of USIs. In addition, BSIs and TSIs reduce the stress concentration and total dislocation on the uninjured contralateral side of the sacrum. This reduces the risk of an adjacent fracture on the uninjured side of the sacrum and fracture progression and may be associated with a better clinical outcome and faster recovery in the elderly population.

## Figures and Tables

**Table 1:** Spring stiffnesses from previously published studies are shown. ASL: anterior sacroiliac ligament, SPSL: short posterior sacroiliac ligament, LPSL: long posterior sacroiliac ligament, ISL: interosseous sacroiliac ligament, SS: sacrospinous ligament, ST: sacrotuberous ligament, SP: superior pubic ligament, IP: inferior pubic ligament.

	Shi et al. [24]	Yao et al. [23]	Eichenseer et al. [31]
	N/mm	N/mm	N/mm Defined by the Percentage Elongation
ASL	700	18.9	39–103
SPSL	400	21.0	200–525
LPSL	1.000	21.0	29–75
ISL	2.800	22.4	13–34
SS	1.400	12.6	26–68
ST	1.500	22.5	17–45
SP	500	12.0	/
IP	500	12.0	/

**Table 2:** The distance of the fracture gap in the lateral–medial direction (mm) is shown depending on the load application and fixation technique.

Load Application	USI	BSI	TSI
Bipedal stance			
Posterior pelvic ring	1.84	1.21	0.69
Anterior pelvic ring	4.13	1.53	−0.20
Right one-legged stance			
Posterior pelvic ring	3.22	2.05	0.86
Anterior pelvic ring	5.63	3.31	−1.19
Left one-legged stance			
Posterior pelvic ring	0.01	0	0.05
Anterior pelvic ring	3.01	0.13	0.13

**Table 3:** Observed stress in the lateral–medial direction (MPa) is shown depending on the load application and fixation technique.

Load Application	USI	BSI	TSI
Bipedal stance			
S1 right side	9.95	7.22	5.23
S1 left side	7.55	4.26	4.88
S2 right side	5.11	2.51	3.27
S2 left side	4.76	2.49	3.26
Right one-legged stance			
S1 right side	19.99	16.23	9.27
S1 left side	−1.37	−0.56	0.28
S2 right side	8.77	6.77	4.92
S2 left side	1.33	0.41	2.08
Left one-legged stance			
S1 right side	−0.52	−0.42	0.20
S1 left side	15.71	9.98	8.91
S2 right side	1.09	0.04	0.67
S2 left side	10.58	9.28	7.94

## Data Availability

All data are available on request.
